# Volatile Organic Compounds From *Lysobacter capsici* AZ78 as Potential Candidates for Biological Control of Soilborne Plant Pathogens

**DOI:** 10.3389/fmicb.2020.01748

**Published:** 2020-08-07

**Authors:** Anthi Vlassi, Andrea Nesler, Michele Perazzolli, Valentina Lazazzara, Christoph Büschl, Alexandra Parich, Gerardo Puopolo, Rainer Schuhmacher

**Affiliations:** ^1^Department of Agrobiotechnology (IFA-Tulln), Institute of Bioanalytics and Agro-Metabolomics, University of Natural Resources and Life Sciences Vienna (BOKU), Tulln, Austria; ^2^Bi-PA nv (Biological Products for Agriculture), Londerzeel, Belgium; ^3^Center of Agriculture, Food, Environment, University of Trento, San Michele all’Adige, Italy; ^4^Department of Sustainable Agro-Ecosystems and Bioresources, Research and Innovation Centre, Fondazione Edmund Mach, San Michele all’Adige, Italy

**Keywords:** *Lysobacter capsici* AZ78, VOC, GC-MS, pyrazine, biological control, soilborne plant pathogens

## Abstract

The genus *Lysobacter* includes several bacterial species which show potential for being used in biological control of plant diseases. It was shown recently that several *Lysobacter* type strains produce volatile organic compounds (VOCs) which controlled the growth of *Phytophthora infestans in vitro* when the bacteria were grown on a protein rich medium. In the present study, *Lysobacter capsici* AZ78 (AZ78) has been tested for its potential to produce VOCs that may contribute to the bioactivity against soilborne plant pathogens. To this end, split Petri dish assays of bacterial cultures have been combined with GC-MS measurements with the aim to reveal the identity of the VOCs which inhibit the growth of *Pythium ultimum Rhizoctonia solani*, and *Sclerotinia minor*. While AZ78 completely suppressed the growth of *P. ultimum* and *S. minor*, the growth of *R. solani* was still reduced significantly. The GC-MS analysis revealed 22 VOCs to be produced by AZ78, the majority of which were (putatively) identified as mono- and dialkylated methoxypyrazines. Based on additional cultivation and GC-MS experiments, 2,5-dimethylpyrazine, 2-ethyl-3-methoxypyrazine and 2-isopropyl-3-methoxypyrazine were selected as presumable bioactive compounds. Further bioassays employing indirect exposure to standard solutions (1–10 mg per Petri dish) of the synthetic compounds via the gas phase, revealed that each of these pyrazines was able to suppress the growth of the pathogens under investigation. The results of this study highlight the possible future implementation of pyrazine derivatives in the control of soilborne plant diseases and further support the biocontrol potential of *L. capsici* AZ78.

## Introduction

The recent years, the scientific community is increasingly questioning the use of synthetic chemical pesticides in plant disease management, as their impact on human health and the environment are coming to surface (Alavanja et al., 2004; Köhler and Triebskorn, 2013). As a result, regulations become stricter and some synthetic chemical pesticides have been already banned, as in the case of methyl bromide (2011/120/EU). This pesticide was constantly used in soil fumigation for the control of the soilborne phytopathogenic microorganisms *Pythium ultimum*, *Rhizoctonia solani* and *Sclerotinia minor*, responsible for a plethora of commercially significant plant diseases (Ah-Fong et al., 2017; Verwaaijen et al., 2017; Crutcher et al., 2018). Considering their life cycle, it is undeniable that soil fumigation is an effective way for managing soilborne phytopathogenic microorganisms. However, as the use of several conventional chemical fumigants has been banned, alternative compounds are needed to be used in soil fumigation.

Recently, microbial volatile organic compounds (VOCs) have come into the limelight as potential candidates to substitute the outlawed chemical fumigants (De Boer et al., 2019). Indeed, their physiochemical characteristics such as low boiling point, high vapor pressure and low molecular weight (<300 Da) (Piechulla et al., 2017), their activity in both gas and liquid phases and ability to re-evaporate after diffusing through water-filled pores, allow microbial VOCs to move easily through the soil pores network (Effmert et al., 2012). Another aspect is that microbial VOCs exhibit various biological properties beneficial for plant health, like enhancing plant growth, inducing resistance against abiotic and biotic stress and inhibiting spore germination and mycelial growth of plant pathogens (Bitas et al., 2013). All these properties and characteristics point to the exploitation of microbial VOCs in soil fumigation belowground.

A growing number of studies that bring attention to the practical application of bacterial VOCs in agriculture have emerged. Bacteria residing in agricultural soils release VOCs belonging to the classes of terpenes, pyrazines, sulfur compounds and other nitrogen containing metabolites that have been described to show antifungal activities (Tyc et al., 2017). VOCs such as dimethyl disulfide and 2,3-butanediol emitted from *Bacillus* sp. and *Enterobacter* sp. have for example been applied as soil treatments in open field and greenhouse experiments, that protected tobacco (*Nicotiana benthamiana* D.) and maize (*Zea mays* L.) plants against several pathogenic fungi (*Botrytis cinerea*, *Colletotrichum heterostrophus*, and *Setosphaeria turcica*) (Chung et al., 2016). In particular, dimethyl disulfide, that has been widely studied for its positive effects on plant health (Groenhagen et al., 2013; Popova et al., 2014; Giorgio et al., 2015), has already been commercialized as a soil pre-fumigant against diseases caused by nematodes and soilborne plant pathogens (Paladin, Arkema, Colombes, France).

The genus *Lysobacter* (Christensen and Cook, 1978) consists of bacteria isolated from various environments like freshwater, agricultural soils and plant rhizosphere (Reichenbach, 2006; Puopolo et al., 2018). Notably, the occurrence of bacterial strains belonging to the species *L. antibioticus*, *L. capsici*, and *L. gummosus* in clay agricultural soils, was related to the disease suppressiveness against *R. solani* (Postma et al., 2008, 2010). Several *Lysobacter* sp. have been employed as biocontrol agents, including *L. capsici* AZ78 (AZ78), which was isolated from the rhizosphere of tobacco and has been shown to produce a variety of soluble secondary metabolites with detrimental effects on several plant pathogens (Puopolo et al., 2014, 2016). *Lysobacter* spp. may control phytopathogenic microorganisms using several other modes of actions such as lytic enzymes and by the ability to prey upon phytopathogenic microorganisms (Nakayama et al., 1999; Folman et al., 2004; Tomada et al., 2017).

The involvement of the release of bioactive VOCs in the ability of *Lysobacter* spp. to control soilborne phytopathogenic microorganisms did not receive particular attention so far. However, it was reported that VOCs produced by *Lysobacter* and other bacterial species were positively correlated to the natural ability of certain soils to suppress the growth of phytopathogenic fungi (Zou et al., 2007). Looking specifically at the antagonism against *Phytophthora infestans*, four *Lysobacter* spp., including *L. capsici* type strain, were shown to release VOCs with toxic activity against the pathogen (Lazazzara et al., 2017).

Based on this body of knowledge, in this study we aimed at exploring the involvement of VOCs released by AZ78 in the inhibition of three soilborne plant pathogens, namely *P. ultimum, R. solani*, and *S. minor* and identifying candidate VOCs for the development of novel biopesticides in the future.

## Materials and Methods

### Microorganisms, Growth Media, and Conditions

AZ78 was stored in 40% glycerol stocks at −80°C and routinely grown on Luria-Bertani Agar (LBA, Sigma-Aldrich, St. Louis, MO, United States; Oxoid, Basingstoke, United Kingdom) in 90 mm Petri dishes (Sarstedt, Nümbrecht, Germany or VWR International, Leuven, Belgium) for 72 h at 27°C. In all the experiments, AZ78 cell suspensions were prepared after 72 h growth at 27°C and by scraping the AZ78 colonies from the LBA surfaces using sterile 10 μL loops. Loopful of AZ78 cells were then thoroughly suspended in 10 mL of sterile NaCl solution (0.85%, w/v) and the absorbance was adjusted to an optical density of 0.1 at 600 nm (A_*OD*__600_) corresponding to 1 × 10^8^ cells/mL (Puopolo et al., 2016), using a spectrophotometer (UV-2450, Shimadzu, Kyoto, Japan). The soilborne plant pathogenic microorganisms *P. ultimum*, *R. solani*, and *S. minor*, from the in-house culture collection of Fondazione Edmund Mach, were regularly grown on potato dextrose agar (PDA, Oxoid) at 25°C in the dark.

### Inhibition Assay of *Lysobacter capsici* AZ78 VOCs Against Soilborne Plant Pathogens

Split Petri dishes (Sarstedt, diameter 92 mm), with ventilation cams and two physically separated compartments were used. 5 mL of PDA medium were poured in one compartment and Nutrient Agar (NA, Oxoid) in the other. After solidification of the media, 50 μL of AZ78 cell suspension were spread on NA using sterile spatulas. Subsequently, Petri dishes were sealed with Parafilm (Bemis, Neenah, United States) and incubated for 72 h at 25°C. Petri dishes containing uninoculated NA medium were used as control. In all the experiments, a plug (5 mm in diameter) was cut from the border of 7 days old fungal or oomycete colony using a sterile cork borer. Subsequently, the mycelium plugs were placed on PDA-containing side and the Petri dishes were incubated at 25°C in the dark. After 96 h post-inoculation (hpi), the mycelial growth of the tested pathogens was determined by measuring the mycelium colony diameter (mm) parallel to the separating barrier of the two compartments. It is worth noting that the 96 hpi time point of the measurements corresponded to a total AZ78 incubation time of 168 h, as AZ78 was incubated for 72 h prior to pathogens inoculation. Thus, 168 h was subsequently chosen as one of the sampling time points for the analysis of AZ78 VOCs profile described below. Four replicates (Petri dishes) were analyzed for each treatment and the experiment was carried out twice.

### Headspace Gas Chromatography-Mass Spectrometry Analysis of Volatile Organic Compounds Produced by *Lysobacter capsici* AZ78

#### Sample Preparation of Living Cultures for Gas Chromatography-Mass Spectrometry

Headspace (HS) vials (20 mL, La-Pha-Pack, Langerwehe, Germany) were filled with 5 mL of NA and were placed in a slanted position under a laminar flow overnight. The vials were left open to avoid water condensation. The slanted position was chosen for creating more surface area for the bacteria to grow on. Subsequently, a 50 μL aliquot of bacterial suspension (1 × 10^8^ cells/mL) was added to the HS vials, the bacteria were spread on the surface of the medium with the help of a sterile loop and the vials were left to dry under a laminar flow for 1 h at room temperature. The HS vials were then sealed with sterile metal caps containing 1.3 mm-silicone/PTFE septa (La-Pha-Pack) and incubated at 28°C for 120 h or 168 h before GC-MS measurement. Empty HS vials and HS vials filled with NA in absence of AZ78 were used as control. Six to seven replicates (HS vials) were analyzed for each treatment in a randomized block design and the experiment was carried out twice.

#### Preparation of Potato Dextrose Agar Samples for Gas Chromatography-Mass Spectrometry

To test whether during cultivation on NA, AZ78 VOCs are absorbed into the PDA medium, split Petri dishes (Sarstedt, diameter 92 mm) containing 5 mL NA and 5 mL PDA medium respectively, were used. Bacteria were inoculated in the compartment with NA (50 μL of a 1 × 10^8^ cells/mL suspension), the Petri dishes were sealed with Parafilm (Bemis) and incubated at 27°C for 168 h. The cultivation period of 168 h was used since pilot studies using headspace vials revealed the richest volatile profile of AZ78. PDA and NA, the latter with AZ78 cultures, were then cut into pieces under the laminar flow with the help of a sterile spatula. The pieces of each medium were placed into separate sterile HS vials, which were sealed with sterile metal caps having 1.3 mm silicone/PTFE septa and the VOC profiles were measured by GC-MS according to the parameters specified below. Empty HS vials and HS vials with uninoculated NA and PDA pieces respectively, were used as controls. Four or five replicates (HS vials) were analyzed for each treatment in a randomized block design and the experiment was carried out twice.

#### Gas Chromatography-Mass Spectrometry Analysis of *Lysobacter capsici* AZ78

All analyses were performed with an Agilent 6890N gas chromatograph (GC, Agilent Technologies, CA, United States) coupled with an Agilent 5975B quadrupole mass selective detector (MSD, Agilent). The GC-MS was additionally equipped with a multi-purpose autosampler (MPS 2XL, Gerstel, Mülheim an der Ruhr, Germany), a dynamic headspace system (DHS, Gerstel), a thermal desorption unit (TDU, Gerstel) and a cooled injection system (CIS, Gerstel) unit. After initial optimization of the parameters for extraction of VOCs, transfer of the analytes to the TDU, desorption and cold injection, the following settings were chosen for the DHS: samples were pre-incubated at 27°C for 15 min and simultaneously agitated at 1000 rpm. VOCs were then collected dynamically (N_2_ purge flow 100 or 50 mL/min to a final volume of 500 mL) onto a 2 cm TENAX trap (Buchem, Apeldoorn, Netherlands) at 30°C. Subsequently, the loaded trap was dried at 27°C in a N_2_ gas stream (100 or 50 mL/min to a final volume of 800 mL) and transferred to the TDU. Thermal desorption of the analytes into the CIS (−150°C) was achieved by heating from 30 to 230°C at a rate of 60°C/min (hold time 5 min). A liner-in-liner setup was operated with a transfer temperature of 250°C in splitless mode. The temperature was increased from −150°C to 250°C at a rate of 2°C/s (hold time 6 min). Chromatographic separation was carried out on a non-polar HP-5MS (5% phenyl- 95% methylsiloxane) column (30 m × 0.25 mm × 0.25 μm). The oven program was set to 35°C for 2 min, then the temperature was raised to 200°C at 5°C/min (hold time 1 min) and increased from 200 to 250°C at 20°C/min (hold time 5 min). The carrier gas was He at a constant flow rate of 1 mL/min. The temperature of the transfer line was set to 270°C, the ion source to 230°C and quadrupole to 150°C. Electron ionization was achieved at 70 eV and a scan range of *m/z* 15–500 was used.

Data analysis was carried out using the open-source software MetaboliteDetector (Hiller et al., 2009), version-3.1.^1^. The following parameter settings were used: peak threshold 5, peak height 5, bins/scan 10 and deconvolution width (scan) 5. For identification and annotation of the detected compounds, a similarity score was calculated by the MetaboliteDetector, which combines both retention index RI as well as the similarity of the compound mass spectra. For the similarity score, the threshold was set to ≥ 0.8 with a ΔRI < 5. While identification was based on the comparison with an in-house library of authentic standards measured under the same chromatographic conditions, compound annotation relied on comparison with entries listed in the NIST 14 library (National Institute of Standards and Technology, United States)^2^. When no spectral information was found in the NIST library, the information from literature (Gallois and Grimont, 1985; Dickschat et al., 2005) was used for the annotation of the respective compound. The RI was automatically calculated by the software by comparing the experimental retention time in relation to those of a series of n-alkanes (C8–C25) analyzed in the same measurement sequence. The data were curated by manual inspection and the detected compounds were assigned identification levels according to the criteria described by Blazenovic et al. (2018) where confidence level (C.L.) 1: RI and MS spectrum matched to authentic reference standard, C.L. 2: RI and MS spectrum matched to literature and NIST 14 library respectively, C.L. 3: possible structure (class), the most likely structure is given based on comparison with literature spectra.

### Comparative Assessment of Growth of *Lysobacter capsici* AZ78 Cultures After Profiling of Volatile Organic Compounds

In order to ensure uniform bacterial growth in the various replicates of AZ78-inoculated HS vials, the OD_600_ value of each replicate was monitored after GC-MS analysis. Briefly, the HS vial was flooded with 5 mL of sterile NaCl solution (0.85%, w/v) and bacterial cells were scraped from the medium surface by vortexing. The cell concentration of the resulting cell suspension was assessed by recording the OD_600_ of a 1 mL-aliquot using a spectrophotometer (Shimadzu) and a micro cuvette with 1 cm path length (1 × 1 cm).

### Inhibition Assay Against Soilborne Plant Pathogens Using Synthetic VOCs

Three out of 22 detected VOCs, namely 2,5-dimethylpyrazine (4), 2-ethyl-3-methoxypyrazine (9) and 2-isopropyl-3-methoxypyrazine (10), were selected and used as synthetic compounds (Merck, Sigma-Aldrich, Darmstadt, Germany) for inhibition tests, according to their presence in AZ78-inoculated HS vials and PDA medium from split Petri dishes with AZ78-inoculated NA medium. Split Petri dishes with two compartments were used as follows: filter paper (90 mm, VWR) was cut in half and placed in one side of the Petri dish, while 5 mL of PDA medium was poured in the other side. The compounds were dissolved in ethanol (Sigma-Aldrich) at concentrations of 500, 375, 250, 125, and 50 g/L assuming the complete VOC dissolution and 20 μL aliquots were added to the filter paper of each Petri dish resulting in dosages of 10.0, 7.5, 5.0, 2.5, and 1.0 mg/Petri assuming the complete VOC evaporation from the filter paper, respectively. Split Petri dishes having filter paper treated with 20 μL ethanol were used as control. The Petri dishes were sealed with Parafilm and incubated at 25°C for 72 h to allow VOCs to evaporate into the gas phase of the Petri dish and to absorb into the PDA medium. Subsequently, each plant pathogen (5 mm plug) was inoculated on the PDA compartment (one pathogen per Petri dish) and dishes were sealed with Parafilm and incubated at 25°C. After 48 h incubation, the mycelium colony diameter was measured as reported above and the mycelial growth inhibition was calculated according to the following formula:

$$\begin{array}{l} \text{Mycelial growth inhibition (\%)}=\\ \frac{\text{diameter in untreated control}-\text{diameter in treated dishes}}{\text{diameter in untreated control}}\times 100 \end{array}$$

Four or five replicates (Petri dishes) were analyzed per treatment and the experiment was carried out twice.

### Statistical Analysis

All experiments were performed twice and six to seven replicates were used in the case of GC-MS measurements of HS vials, while four to five replicates were used for GC-MS measurements of Petri dishes and in the microbiological assays using Petri dishes. For VOC assignment in AZ78 and PDA samples, we only considered compounds that were detected at peak area levels > 10 × that of control samples. Initially, growth diameters of pathogens, resulting from the inhibition assays with synthetic VOCs, were evaluated separately for each experiment for normal distribution (Shapiro-Wilk test, *p* > 0.05) and homogeneity of variance (Levene’s tests, *p* > 0.05). As both assumptions were met, the data were analyzed using statistical parametric tests. Non-significant differences were found between the two experiments (*p* > 0.05) after two-way analysis of variance (ANOVA) and thus data from the two experimental repetitions were pooled. Subsequently, one-way ANOVA was applied and Tukey’s HSD test (*p* ≤ 0.05) was used as a *post hoc* test to detect significant differences among treatments. In the case of inhibition assays using AZ78, the non-parametric Mann Whitney Test (*p* ≤ 0.05) was applied to estimate differences in the pairwise comparisons between treatments and control samples. The statistical analysis was performed using the IBM SPSS software v. 26 (NY, United States).

## Results

### Inhibitory Effect of *Lysobacter capsici* AZ78 VOCs on Soilborne Plant Pathogens

**FIGURE 1:**
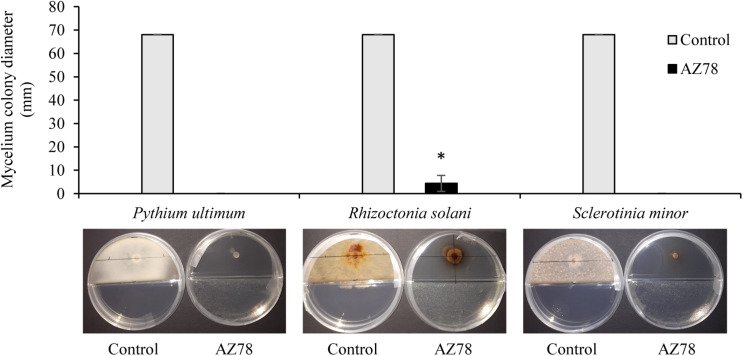
Effect of *Lysobacter capsici* AZ78 volatile organic compounds (VOCs) on the radial growth of soilborne plant pathogens. The colony diameter was measured 96 h after incubation of plant pathogen in split Petri dishes with AZ78 growing on NA medium in the physically separated compartment. Presented values are means ± standard error pooled from two experiments of four replicates each. Bar with asterisk differs significantly from control according to Mann–Whitney test (*p* ≤ 0.05). Control: Petri dishes not inoculated with AZ78.

At 96 hpi, all three soilborne plant pathogens covered the total surface of the PDA growth medium when grown in absence of *L. capsici* AZ78 in split Petri dishes. In contrast, the presence of AZ78 grown on NA caused a drastic inhibition of the mycelial growth of the plant pathogenic microorganisms tested. Particularly, *P. ultimum* and *S. minor* mycelium development was totally inhibited as no growth was observed (Figure 1), while *Rhizoctonia solani* growth was significantly inhibited by AZ78 VOCs, compared to the control (Figure 1).

### The VOC Profile of *Lysobacter capsici* AZ78 and Presumably Bioactive Constituents

The dynamic headspace-thermodesorption-GC-MS (DHS-TD-GC-MS) analysis resulted in 22 VOCs that were consistently found in AZ78 samples 120 or 168 h after incubation in HS vials with NA medium (Table 1). Pyrazines clearly dominated the bacterial profiles at both time points, with structures of monoalkyl-methoxypyrazines and dialkyl-methoxypyrazines to be the most abundant. In total, five monoalkyl-methoxypyrazines (6, 9, 10, 13, and 14) were identified (C.L. 1) having methyl-, ethyl-, isopropyl-, sec- butyl-, and isobutyl- substitutions, respectively. With respect to dialkylated pyrazines, four methyl- derivatives of the respective ethyl-, isopropyl-, sec-butyl- and isobutyl-methoxypyrazines (11, 12, 16, and 18) were annotated (C.L. 3 and 2). Two more pyrazines, having a diisopropyl-methoxy structure (20 and 21) were annotated (C.L. 3), as well. One dialkylpyrazine was detected and identified (C.L. 1) as 2,5-dimethylpyrazine (4). Moreover, four more metabolites were assigned to the compound class of pyrazines (15, 17, 19, and 22) and were annotated (C.L. 3) as dimethyl- derivatives of the ethyl-, isopropyl-, and *sec*-butyl-methoxypyrazines structures, respectively. The mass spectra of the pyrazines (11), (15), (17), (19), and (22) could not be matched to any spectra registered in NIST library or reported in literature. They shared ion fragments though, with the rest monoalkyl- and dialkyl-methoxypyrazines found in the VOC blend of AZ78. Thus, the classification of these compounds as pyrazines is based on the analysis of their experimental GC-MS spectra (Supplementary Table S1). In addition to the numerous pyrazines, the two alcohols 3-methyl-1-butanol (1) and 2-ethyl-1-hexanol (7) were identified (C.L. 1), as well. Furthermore, two esters were detected, of which methyl 2-methylbutanoate (3) structure was confirmed by authentic reference compound (C.L. 1), while methyl-2-ehtylhexanoate (8) structure was annotated (C.L. 2). The 2-methyl-3-(methylthio)-furan (5), and pyrrole (2), were identified (C.L. 1) as further constituents of the VOC mixture produced by AZ78.

**TABLE 1:** The volatile profile of *Lysobacter capsici* AZ78.

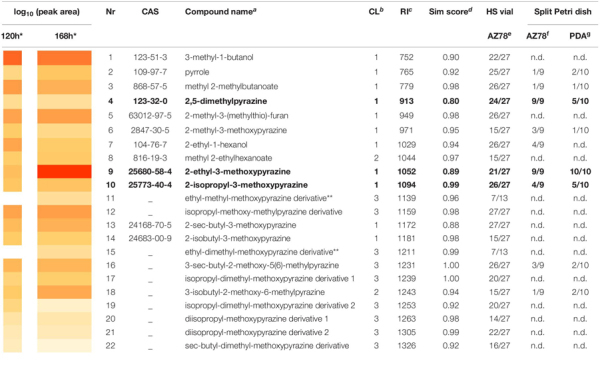

**Compounds listed were at least with 10 x more abundance in samples compare to control. ^∗^Heatmap of log_10_(average peak area) of compounds at 120 h and 168 h measurements of AZ78-inoculated HS vials with NA medium. ^∗∗^Compounds only detected at 168 h measurements of AZ78-inoculated HS vials with NA medium. ^*a*^Compounds identified or annotated using Metabolite Detector Software (Hiller et al., 2009), by comparing both spectrum and RI to those of an in-house library compound entry. ^*b*^Confidence levels of compound annotation assigned according to Blazenovic et al. (2018): 1 = confident 2D structure by comparison of spectrum and RI to standard compounds analyzed under the same chromatographic conditions, 2 = probable structure by comparison of spectrum and RI to those of NIST library or literature, 3 = possible structure or compound class by comparison of spectrum to those of known compounds in literature. ^*c*^Average retention index of six to seven replicates of AZ78-inoculated HS vials with NA medium from 120 h and 168 h measurements, as calculated from the experimental retention time of each VOC in relation to those of a series of n-alkanes (C8–C25) analyzed under the same chromatographic conditions. ^*d*^Average similarity score of six to seven replicates of AZ78-inoculated HS vials with NA medium from 120 h and 168 h measurements as calculated by Metabolite Detector software for each VOC by comparing both RI and spectrum to those of an in-house library compound entry. ^*e,f,g*^Number of sample replicates the VOC was detected in, to the total replicate number measured in ^*e*^ AZ78-inoculated HS vials with NA medium, ^*f*^AZ78-inoculated NA medium from split Petri dishes and ^*g*^PDA medium samples from split Petri dishes with AZ78-inoculated NA medium in a separate compartment. n.d = Not detected. in bold = Compounds selected to be used in bioassays against soilborne plant pathogens.**

Comparing the two time points, the VOC profiles exhibited differences in both relative abundances (expressed as peak area) as well as type of metabolites. At 120 h, the most abundant compound was 3-methyl-1-butanol (1), while 2-ethyl-3-methoxypyrazine (9) was detected in the highest amount 168 h after incubation, with (1) being the second most abundant compound at 168 h. The two pyrazine derivatives 11 and 15 were solely detected at 168 h of incubation. In general, most of the compounds (4, 5, 8, 10, 12, 14, 16, 17, 20, and 21) were produced in similar amounts in both time points.

To investigate which VOCs potentially contributed to the inhibitory activity of AZ78 against the soilborne plant pathogens, we further measured the volatile profiles of AZ78-inoculated NA medium and PDA medium of the same split Petri dish. At 168 h time point, nine compounds (2, 3, 4, 6, 7, 9, 10, 16, and 18) were detected above AZ78-inoculated NA medium of the split Petri dish, all of which were also previously detected in the volatile profile of AZ78-inoculated NA medium of the HS vials. Except for 2-ethyl hexanol (7), all these compounds were also detected in the headspace of PDA medium of the same split Petri dish where AZ78 was inoculated on NA. Most of the compounds that were found above the PDA medium belonged to the pyrazines. Interestingly, compounds like (1), (5), (12), and (13) that were constantly detected in high amounts throughout the measurements of AZ78 in HS vials (120 h or 168 h), were not detected in either AZ78 or PDA samples from Petri dishes. The compounds (4), (9), and (10) were most consistent, being detected in at least half of the measured replicates. As these three pyrazines (4, 9, and 10) were also constituents of the AZ78 profile measured in HS vials (see also Supplementary Table S2), they were selected to further test their inhibitory activity against the soilborne plant pathogenic microorganisms.

### Inhibitory Effect of Synthetic VOCs of *Lysobacter capsici* AZ78 on the Growth of Soilborne Plant Pathogens

**FIGURE 2:**
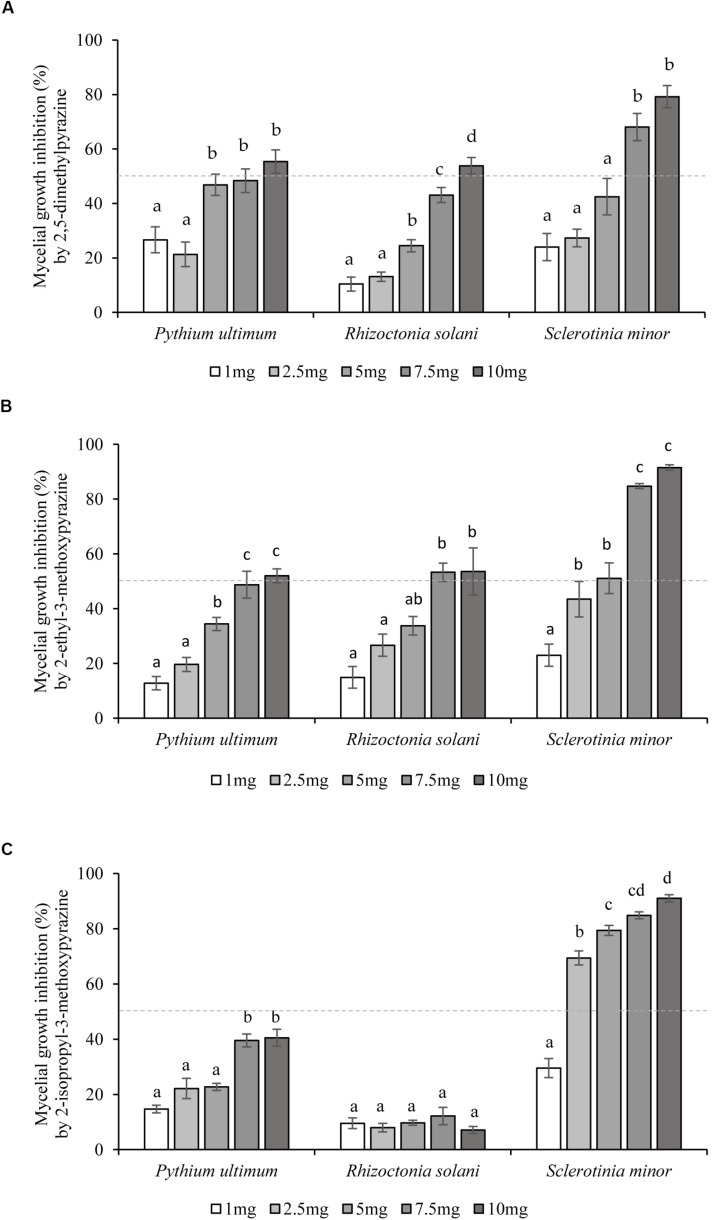
Inhibition of soilborne plant pathogens growth by single synthetic *Lysobacter capsici* AZ78 VOCs. The inhibition effect of five concentrations (1–10 mg/Petri dish) of each synthetic VOC: **(A)** 2,5-dimethylpyrazine, **(B)** 2-ethyl-3-methoxypyrazine, and **(C)** 2-isopropyl-3-methoxypyrazine, was assessed 48 h after simultaneous incubation with the soilborne plant pathogens. The percentage of inhibition was calculated by comparing the radial growth of treated pathogens to the radial growth of pathogens from untreated Petri dishes used as control. Presented values for each treatment are means ± standard error pooled from two experiments each having six or seven replicates. Dotted line indicates the 50% inhibition level. For each soilborne plant pathogen, bars with different letters were found to be significantly different according to Tukey’s HSD test (*p* ≤ 0.05).

2,5-Dimethylpyrazine (4), 2-ethyl-3-methoxypyrazine (9), and 2-isopropyl-3-methoxypyrazine (10), were tested as synthetic compounds in assays against soilborne plant pathogens. All three VOCs were effective against *P. ultimum*, *R. solani*, and *S. minor* at concentrations ranging from 1.0 to 10.0 mg/Petri at 48 hpi (Figures 2A–C). In terms of selectivity and minimum dosage, 2-ethyl-3-methoxypyrazine (9) was the most active of the tested compounds, inhibiting *R. solani* by 53.3 ± 3.4% at 7.5 mg/Petri dosage, *S. minor* by 51.1 ± 5.6% at 5.0 mg/Petri and *P. ultimum* by 52.0 ± 2.6% at 10.0 mg/Petri (Table 2 and Figure 2B). The second most active compound was 2,5-dimethylpyrazine (4) that inhibited the growth of *P. ultimum* and *R. solani* by more than 50% at 10 mg/Petri and *S. minor* at 7.5 mg/Petri dosage (Table 2 and Figure 2A). Moreover, 2-isopropyl-3-methoxypyrazine (10) was the most efficient VOC in limiting the growth of *S. minor*, as a dosage as low as 2.5 mg/Petri attenuated the growth of the pathogen by 69.4 ± 2.6% (Table 2 and Figure 2C). On the other hand, *P. ultimum* growth was inhibited by less than 50% by 2-isopropyl-3-methoxypyrazine (10). Additionally, this compound was also the least effective VOC against *R. solani* since this pathogen was only inhibited by around 10% (Table 2 and Figure 2C).

**TABLE 2:** Minimum concentration of single synthetic *Lysobacter capsici* AZ78 VOCs for the inhibition of soilborne plant pathogens growth.

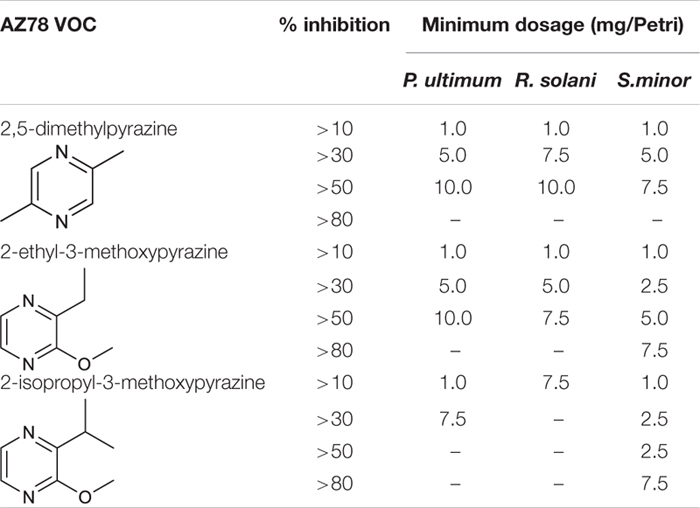

**The respective chemical structure of each VOC is shown.**

## Discussion

AZ78 has been shown to share all the physiological traits useful to survive in plant rhizosphere and successfully compete with plant pathogens through the production of non-volatile secondary metabolites (Puopolo et al., 2016; Brescia et al., 2020). Recently, Lazazzara et al. (2017) showed that type strains of *Lysobacter* spp. could produce VOCs, like pyrazines and pyrrole, that further contribute to the bioactivity of these strains against *Ph. infestans*. Based on these results, we investigated the ability of AZ78 to produce VOCs, hypothesizing that these compounds may inhibit the growth of soilborne plant pathogens *in vitro*.

In agreement with our initial assumption, the growth of the three pathogens investigated was totally (*P. ultimum, S. minor*) or largely (*R. solani*) inhibited by AZ78 VOCs that can diffuse in the headspace of the split Petri dish. The DHS-TD-GC-MS analysis revealed that AZ78 is able to emit a relevant number of VOCs that may have a toxic effect against the plant pathogens tested, as for example the alcohols 3-methyl-butanol (1) and 2-ethyl-1-hexanol (7). Indeed, (1) has been found in bacteria, yeast and endophytic fungi and inhibited the growth of various phytopathogenic fungi and oomycetes (Strobel et al., 2001; Syed-Ab-Rahman et al., 2019). Similarly, (7) was found in the volatile profile of several *Pseudomonas* spp. and when tested as synthetic compound, completely inhibited mycelial growth and sclerotia germination of *S. sclerotiorum* (Fernando et al., 2005).

The compound class of pyrazines dominated the AZ78 VOC profile in both 120 and 168 h old cultures. Pyrazine derivatives exhibit several activities of pharmacological interest including antibacterial, antifungal, antidiabetic, anticancer, antiviral, and analgesic activity (Dolezal and Zitko, 2015) and microorganisms, including bacteria and fungi, represent a major source for biosynthesis of pyrazines (Rajini et al., 2011). Various mono- and dialkylpyrazines produced by soil bacteria significantly inhibited the growth of a wide variety of phytopathogenic fungi and oomycetes *in vitro* (Haidar et al., 2016; Mülner et al., 2019). Regarding their mode of action, studies testing the activity of 2,5-diisopropylpyrazine against *Escherichia coli*, showed that the compound caused DNA damage at high concentrations, while at lower levels, cell-wall damage was observed. Similar mechanisms could apply for phytopathogenic fungi, as the growth of *Fusarium culmorum* and *R. solani* strains was also strongly attenuated by this pyrazine (Janssens et al., 2019).

Regarding the type of pyrazines produced by AZ78, the methoxypyrazines with various mono- and dialkylated derivatives was the dominant group of VOCs. The monoalkyl-methoxypyrazines (6, 9, 10, 13, and 14) identified in the profile, are widely distributed metabolites of plants, insects, fungi and bacteria. For instance, they are known semiochemicals of ladybird beetle (*Harmonia axyridis*) and their occurrence in insects can be related to bacterial metabolism (Schmidtberg et al., 2019). Their biosynthesis in bacteria is proposed to originate from condensation of amino acids like valine or isoleucine with glycine (Cheng et al., 1991; Bungert et al., 2001) and their production has been already described in *Chondromyces crocatus*, *Halomonas venusta*, and *P. perolens* (Cheng et al., 1991; Bungert et al., 2001; Schulz et al., 2004). AZ78 additionally produced several dialkyl-methoxypyrazines (12, 16, 18, 20, and 21) that are rarely reported in bacteria (Chatonnet et al., 2010). 3-Isobutyl-2-methoxy-6-methylpyrazine was for the first time reported as a natural product of *Serratia odorifera* VOC blend by Gallois and Grimont (1985), while 2-isobutyl-3-methoxy-5-methylpyrazine was formerly described for the myxobacterium *C. crocatus* (Dickschat et al., 2005). The compound (18) found in AZ78 VOCs mixture was annotated as 3-isobutyl-2-methoxy-6-methylpyrazine. All other dialkyl-methoxypyrazines detected in the VOC profile of AZ78 were annotated based on the similarity of their mass spectra and RI values with the respective data reported in the two above studies. From the dialkylpyrazine group, 2,5-dimethylpyrazine (4) was confirmed by authentic standards. This metabolite is widespread in nature and is produced by a vast number of bacteria (Schulz and Dickschat, 2007). Although it is also reported to be present in the media used for cultivation or formed during heating or autoclaving of growth media (Schulz and Dickschat, 2007), in our experiments it was either not detected in the medium, or at levels less than 1/10 compared to the bacterial samples.

A relevant number of *in vitro* studies dedicated to the analysis of bacterial VOCs and their bioactivity, concentrate on the compounds emitted directly from the bacterial culture under investigation. We decided to go a step further and focused our attention on the VOCs accumulated on the growth medium which had been exposed to VOC mixtures produced by bacteria without direct contact to the bacterial culture itself. To achieve this, we analyzed the VOCs present above PDA medium, which was physically separated from the growing AZ78 cells, hypothesizing that these VOCs are more likely to contribute to AZ78 inhibitory activity.

Several compounds detected in the VOC profile of AZ78 were identified in the physically separated PDA medium of the same Petri dish. 2,5-Dimethylpyrazine (4), 2-ethyl-3-methoxypyrazine (9), and 2-isopropyl-3-methoxypyrazine (10) were the compounds more consistently detected in PDA medium. We were able to determine the amounts of (4, 9, 10) by preparing standards of each VOC and measuring in parallel with the samples by GC-MS. Comparison of peak areas between standards and pyrazines in samples revealed that concentration levels corresponded to amounts of a few ng per sample of AZ78 cultures and PDA medium (Supplementary Table S2). Based on these results, it is conceivable that AZ78 emits these VOCs and that they accumulate on the surface of PDA medium where they might get in physical contact with the cells of the plant pathogens tested.

As (4, 9, 10) were the most predominant VOCs found, they were evaluated for their inhibitory activity against *P*. *ultimum*, *R*. *solani*, and *S*. *minor.* Interestingly, these VOCs inhibited the mycelial growth of each of the plant pathogenic microorganisms at different concentrations. Overall, *S. minor* was the most sensitive pathogen, as it was the only one inhibited by more than 80% after application of 7.5 mg/Petri of both 2-ethyl-3-methoxypyrazine (9) or 2-isopropyl-3-methoxypyrazine (10) and by more than 50% with 7.5 mg/Petri of 2,5-dimethylpyrazine (4). As these pathogens belong to different phyla, it may well be that morphological and biochemical differences are responsible for the different response to the pyrazine tested.

Regarding the tested VOCs, 2-isopropyl-3-methoxypyrazine (10) showed the lowest activity against *R. solani* (about 10% inhibition) whereas 2-ethyl-3-methoxypyrazine (9) was the most active compound, inhibiting the growth of all three pathogens by at least 30% at 2.5–5.0 mg/Petri. This VOC was detected in high amounts at both time points of AZ78 VOC measurements and was the most abundant constituent of the profile at 168 h. To the best of our knowledge, this is the first evidence on the antimicrobial properties of this pyrazine. 2,5-Dimethylpyrazine (4), attenuated (> 30%) the growth of all plant pathogens at the dosage of 7.5 mg/Petri. This VOC may be produced by various soil bacteria and has been shown to inhibit the growth of several diverse plant pathogens (Haidar et al., 2016; Agisha et al., 2019). The broad distribution and activity indicate a possible role of this compound under environmental conditions. Accordingly, Chuankun et al. (2004) found that 2,5-dimethylpyrazine was specifically found in fungistatic soils, or soils that naturally inhibit fungal propagules growth. Thus, it is conceivable that this VOC possesses characteristics that might be exploited for the control of soilborne plant pathogenic microorganisms.

It is worth noting that the inhibition levels observed in bioassays with the synthetic compounds ranged below those exhibited by the VOC bouquet emitted by AZ78, as already reported in other studies (Groenhagen et al., 2013). Thus, we cannot exclude the possible synergistic effect of the various constituents of AZ78 VOC blend on the inhibition of the soilborne plant pathogens and additionally consider the deviations of the *in vitro* conditions from those observed in nature that may lead to differences in VOC production rates. Moreover, it should also be noted that due to the chosen GC-MS approach some volatiles with very low boiling points such as the inorganic NH_3_ or HCN may have been missed during our analysis and therefore could not be considered in the interpretation of activity profiles.

Overall, the present study sheds light on the AZ78 ability to inhibit *in vitro* the growth of soilborne plant pathogenic microorganisms through the emission of a VOC blend, where pyrazines are the most represented class of VOCs. As alkylated pyrazines have been applied in the food industry as disinfectants (Kusstatscher et al., 2017; Schöck et al., 2018) and, additionally, pyrazine derivatives are recognized as safe for human/animal consumption and the environment (Rychen et al., 2017), we feel that our results may support the possible development of selected pyrazines as active ingredients of novel biofumigants against soilborne plant pathogenic microorganisms. Our results suggest that additional compounds and/or factors are involved in the growth inhibition observed in the split Petri dish assays, most probably associated with compounds that cannot easily be measured by the presented DHS-TD-GC-MS approach. Future studies on the inhibitory mechanisms involved and on additional volatiles contributing to AZ78 activity will help to draw a more complete picture of AZ78 volatile-mediated inhibition of soilborne plant pathogenic microorganisms.

## Data Availability Statement

All datasets presented in this study are included in the article/Supplementary Material.

## Author Contributions

AV, VL, MP, GP, and RS conceived the study. AV and VL performed the experiments and analyzed the data. GP, AN, and RS coordinated the experiments and helped to draft the manuscript. AV drafted the manuscript. CB wrote R scripts and analyzed the data. AP contributed to the chemical analysis and authentic reference standard measurements. All authors have read and approved the manuscript before submission.

## Conflict of Interest

AN was employed by Biological Products for Agriculture (Bi-PA). The remaining authors declare that the research was conducted in the absence of any commercial or financial relationships that could be construed as a potential conflict of interest.

## Footnotes

1 http://metabolitedetector.tu-bs.de/

2 http://www.nist.gov
