# Supplementary material for: Volatile Organic Compounds From Lysobacter capsici AZ78 as Potential Candidates for Biological Control of Soilborne Plant Pathogens
Source: Front Microbiol. 2020 Aug 7;11:1748. doi: 10.3389/fmicb.2020.01748 (PMC7427108; doi:10.3389/fmicb.2020.01748)
Supplement: Supplementary file 1 [file Data_Sheet_1.PDF]

Supplementary Table S1. Experimental and literature data of the identified (C.L. 1) or annotated (C.L. 2 and 3) methoxypyrazines found in AZ78 VOCs blend.

Nr	Pyrazine name	C.L. [a]	Exp. RI	Lit. RI	Experimental *m/z* (%) [b]	Literature *m/z* (%)	Lit. source
6	2-methyl-3-methoxypyrazine	1	971	– [c]	124(100) [M$^+$], 109(36), 106(44), 95(32), 81(20), 53(26)	–	–
9	2-ethyl-3-methoxypyrazine	1	1052	–	138(100) [M$^+$], 137(40), 123(15), 120(32), 109(44), 107(24)	–	–
10	2-isopropyl-3-methoxypyrazine	1	1094	–	152(33) [M$^+$], 137(100), 124(20), 105(7), 95(7), 68(4)	–	–
11	ethyl-methyl-methoxypyrazine derivative	3	1139	n.a. [d]	152(100) [M$^+$], 151(53), 137(26), 134(26), 123(26), 119(20)	n.a.	n.a.
12	isopropyl-methoxy-methylpyrazine derivative	3	1159	1149	166(44) [M$^+$], 152(12), 151(100) , 138(36), 119(12), 122(9)	166(40) [M$^+$], 151(100), 138(32), 119(15)	Gallois and Grimont 1985
13	2-*sec*-butyl-3-methoxypyrazine	1	1172	–	166(3) [M$^+$], 151(47), 138(100), 137(30), 124(60), 105(13)	–	–
14	2-isobutyl-3-methoxypyrazine	1	1181	–	166(4) [M$^+$], 151(33), 124(100), 94(22), 81(11), 53(7)	–	–
15	ethyl-dimethyl-methoxypyrazine derivative	3	1211	n.a.	166(100) [M$^+$], 165(45), 151(42), 148(21), 137(18), 123(48)	n.a.	n.a.
16	3-*sec*-butyl-2-methoxy-5(6)-methylpyrazine	2	1231	1221	180(3) [M$^+$], 165(47), 152(100), 151(34), 138(60), 137(17)	180(0.3) [M$^+$], 165(45), 152(100), 151(33), 138(85)	Gallois and Grimont 1985
17	isopropyl-dimethyl-methoxypyrazine derivative 1	3	1239	n.a.	180(39) [M$^+$], 166(10), 165(100), 152(37), 133(10)	n.a.	n.a.
18	3-isobutyl-2-methoxy-6-methylpyrazine	2	1243	1239	180(4) [M$^+$], 165(16), 138(100), 109(10), 108(16), 107(10)	180(0.3) [M$^+$], 165(13), 138(100), 109(12), 108(18), 107(10)	Gallois and Grimont 1985
19	isopropyl-dimethyl-methoxypyrazine derivative 2	3	1253	n.a.	180(33) [M$^+$], 166(13), 165(100), 152(33), 133(6)	n.a.	n.a.
20	diisopropyl-methoxypyrazine derivative 1	3	1263	1287	194 (33) [M$^+$], 179(100), 166(53), 151(13), 136(3)	194 (25) [M$^+$], 179(100), 166(40), 151(10), 136(6), 41(10)	Dickschat et. al., 2005
21	diisopropyl-methoxypyrazine derivative 2	3	1305	1287	194 (26) [M$^+$], 179(100), 166(33), 151(6), 136(6), 41(6)	194 (25) [M$^+$], 179(100), 166(40), 151(10), 136(6), 41(10)	Dickschat et. al., 2005
22	*sec*-butyl-dimethyl-methoxypyrazine derivative	3	1326	n.a.	194 (6) [M$^+$], 179(39), 166(100), 165(45), 152(54), 151(15)	n.a.	n.a.

[a] Confidence levels (C.L.) indicates: 1, confident 2D structure; 2, probable structure and 3, possible structure, according to Blazenovic et al., 2018. [b] Relative ion intensities considering the basic peak as 100%. [c] No literature used; compounds identified using original standards. [d] Not available.

Supplementary Table S2. Abundance of pyrazines (4,9,10) as synthetic and naturally occurring, analyzed in parallel by GC-MS.

		Samples	Syn. Com.	HS vial	Same Petri dish		Synthetic compound (ng/vial)*	
Nr	Name	RI[a]	RI[b]	AZ78 on NA	AZ78 on NA	PDA	10	20
4	2,5-dimethylpyrazine	913	914	$(4.3 \pm 1.9) \times 10^5$#	$(1.4 \pm 0.5) \times 10^5$	$(9.6 \pm 4.7) \times 10^4$	$(3.1 \pm 0.1) \times 10^6$	$(5.2 \pm 0.3) \times 10^6$
9	2-ethyl-3-methoxypyrazine	1052	1053	$(6.8 \pm 1.6) \times 10^6$	$(3.4 \pm 1.3) \times 10^4$	$(6.3 \pm 1.6) \times 10^4$	$(3.1 \pm 0.07) \times 10^6$	$(4.8 \pm 0.2) \times 10^6$
10	2-isopropyl-3-methoxypyrazine	1094	1094	$(2.9 \pm 0.7) \times 10^5$	$(8.9 \pm 3.5) \times 10^3$	$(1.4 \pm 0.4) \times 10^4$	$(5.0 \pm 0.2) \times 10^6$	$(7.7 \pm 0.2) \times 10^6$

*Synthetic pyrazines (Sigma-Aldrich) analyzed in the same sequence in two different concentrations (ng/vial). [a] Average retention index of four to seven replicates of AZ78 and PDA samples and [b] Average retention index of three replicates of each synthetic pyrazine concentration, calculated from the experimental retention time in relation to those of a series of n-alkanes (C8 –C25) analyzed in the same GC-MS sequence. #Mean ± standard error values of peak area from three (synthetic pyrazines) or four to seven replicates from two experimental repetitions are reported for each compound.
